# Signal Transduction and Regulation: Insights into Evolution

**DOI:** 10.1155/2016/8604245

**Published:** 2016-07-20

**Authors:** Song Yi, Sidi Chen, Luoying Zhang, Nidhi Sahni

**Affiliations:** ^1^Department of Genetics, Harvard Medical School, Boston, MA 02115, USA; ^2^Department of Genetics & Systems Biology Institute, Yale Cancer Center and Stem Cell Center, Yale University School of Medicine, West Haven, CT 06516, USA; ^3^College of Life Science & Technology, Huazhong University of Science & Technology, Wuhan, Hubei 430074, China; ^4^Department of Systems Biology, The University of Texas MD Anderson Cancer Center, Houston, TX 77030, USA

Recent advances in modern molecular technology and next-generation sequencing platform have brought in enormous insights into evolution. Although challenges remain, evolutionary research now enters a new era and becomes a hybrid discipline encompassing the fields of molecular biology, genetics and genomics, metabolomics, neuroscience, structural and chemical biology, bioinformatics, proteomics, pharmacology, statistics, and computational biology. The common bond or theme that unifies the various fields is the need to reveal complex genotype-phenotype relationships and their mechanistic regulations, attempting to understand the diversifying functionality in life and ultimately find the underlying causes and effective means of treating disease.

Articles in this special issue cover a broad range of topics related to the evolution of genomes, transcriptomes, proteomes, and interactomes in diverse organisms, including humans.

Signal transduction networks are dynamic and constantly under environmental selection in evolution. This topic is covered in the long review article “Multi-OMICs and Genome Editing Perspectives on Liver Cancer Signaling Networks” by S. Lin et al. This review covers multiple advances in the multi-OMICs and genome editing technologies, now available for use in biomedical and cancer research, and provides new perspectives in targeted therapy and precision medicine. Proteome and interactome networks are also investigated in an accompanying research paper “Positive Selection and Centrality in the Yeast and Fly Protein-Protein Interaction Networks.” S. Chakraborty and D. Alvarez-Ponce study the selective pressures of yeast and fly proteome and show that long-term positive selection has preferentially targeted the periphery of the yeast interactome, while interestingly in flies genes under positive selection encode significantly more connected and central proteins.

Transcriptional and functional regulation and conservation during evolution are touched upon in two other reviews in this special issue. In “Circadian Control of Global Transcription,” S. Li and L. Zhang discuss circadian rhythms through investigating expression of clock-controlled genes (CCGs). They summarize the circadian control of CCG transcription in five model organisms, including cyanobacterium, fungus, plant, fruit fly, and mouse, providing an overview to the function of the circadian clock, as well as its output mechanisms adapted to meet the demands of diverse environmental conditions. In “Roles of Hsp70s in Stress Responses of Microorganisms, Plants, and Animals,” A. Yu et al. review the heat shock protein 70s family members, a class of molecular chaperones that are highly conserved and ubiquitous in organisms ranging from microorganisms to plants and humans. This review provides an overview of the specific roles of Hsp70s in response to stress, particularly abiotic stress, in diverse taxa.

Evolutionary principles and adaptation lessons learned from research have been successfully applied to human disease studies, revealing important insights into potential targets for therapy. In the paper “Clinical End-Points Associated with* Mycobacterium tuberculosis* and Lung Cancer: Implications into Host-Pathogen Interaction and Coevolution,” Y. Tian et al. study the cross-link between pathogens and cancer. They examined the association between the* Mycobacterium tuberculosis* (MTB) and lung cancer and found that 62% of the lung cancer samples are MTB-L positive, suggesting a link between MTB infection and lung cancer development. In another paper “Lost Polarization of Aquaporin4 and Dystroglycan in the Core Lesion after Traumatic Brain Injury Suggests Functional Divergence in Evolution,” H. Liu et al. study the roles of aquaporin4 and dystroglycan in brain edema formation after traumatic brain injury. They found that at an early stage of traumatic brain injury aquaporin4 and dystroglycan maintained the polarized distribution, which were subsequently lost from perivascular end-feet and induced cytotoxic brain edema. In addition, in the study entitled “Identification and Evolutionary Analysis of Potential Candidate Genes in a Human Eating Disorder,” U. Sabbagh et al. set out to find genes linked with eating disorders and associated with both metabolic and neural systems (Night Eating Syndrome, NES). Through a text-based analysis, they identified a number of potential candidate genes including VGF. VGF human polymorphism studies contribute to eating disorders and obesity, suggesting a new approach to connect eGWAS and GWAS in NES patients.

Model systems have been instrumental in elucidating signaling mechanisms underlying biological function, providing interesting cues in evolutionary research areas. In the article “Loss of* flfl* Triggers JNK-Dependent Cell Death in* Drosophila*,” J. Huang and L. Xue study a gene,* falafel *(*flfl*), that encodes a fruit fly* Drosophila* homolog of human SMEK. They performed both gain-of-function and loss-of-function analysis in* Drosophila* and identified* flfl* as a negative regulator of JNK pathway-mediated cell death. In another study “Neurotrophin, p75, and Trk Signaling Module in the Developing Nervous System of the Marine Annelid* Platynereis dumerilii*,” A. Lauri et al. study the evolution of neurotrophic signaling in neuronal development, neural circuit formation, and neuronal plasticity using the worm* Platynereis* system. They discovered and validated nucleotide sequences encoding putative neurotrophic ligands and receptors in two annelids, suggesting roles of these molecules in nervous system and circuit development, as well as their involvement in shaping neural networks during protostome-deuterostome evolution.

The great leap in evolutionary research would not be possible without the development of modern molecular technologies. CRISPR/Cas9 system is a powerful technology to perform genome editing in a variety of cell types. In the paper “A CRISPR-Based Toolbox for Studying T Cell Signal Transduction,” S. Chi et al. adapted CRISPR/Cas9-based tools to study human Jurkat cells. They showed that distinct Cas9 variants, including wild-type Cas9, dCas9-KRAB, and sunCas9 function as expected in different Jurkat cell lines.

With the advent of next-generation sequencing platforms, a large number of genomewide sequencing data become available, which greatly facilitate the evolution research field to promote to a new height. In the paper “Computational Analysis of the Binding Specificities of PH Domains,” Z. Jiang et al. study Pleckstrin homology domain proteins with significant insights into binding specificity and abnormal regulation in disease. In addition, in another report “SimFuse: A Novel Fusion Simulator for RNA Sequencing (RNA-Seq) Data,” Y. Tan et al. developed the SimFuse pipeline to accelerate fusion discovery from RNAseq data. SimFuse utilizes real sequencing data as the fusions' background to closely approximate the distribution of reads from a real sequencing library and uses a reference genome as the template from which to simulate fusions' supporting reads, improving the performance metrics, rigor, and control.

The future evolution researchers will need to be well versed in interdisciplinary fields and have the appropriate background to leverage the biological, clinical, and computational resources necessary to understand their data and track dynamic evolution. The communication among these specialty disciplines, acting in unison, will be critical as we strive to uncover answers underlying the complex and often puzzling molecular events in evolution.

We hope that this special issue would shed light on major advances in the broad area of signal transduction with evolutionary insights and engender novel hypotheses and research innovations to investigate deeper mechanisms and further to engineer signaling circuits for personalized therapeutics in human disease.

